# Peripheral muscarinic receptors mediate the anti-inflammatory effects of auricular acupuncture

**DOI:** 10.1186/1749-8546-6-3

**Published:** 2011-01-21

**Authors:** Wai Yeung Chung, Hong Qi Zhang, Shi Ping Zhang

**Affiliations:** 1 School of Chinese Medicine, Hong Kong Baptist University, Kowloon Tong, Hong Kong, China; 2 School of Chinese Medicine and Health Care, The Chinese University of Hong Kong Tung Wah Group of Hospitals Community College, Homantin, Hong Kong, China

## Abstract

**Background:**

The cholinergic and opioid systems play important roles in modulating inflammation. This study tests whether auricular acupuncture (AA) produces anti-inflammatory effects via opioid and peripheral cholinergic receptors in a rat model.

**Methods:**

Rats were anesthetized with chloral hydrate and inflammation was induced by intraplantar injection of carrageenan. Electroacupuncture was performed at auricular points bilaterally. The severity of inflammation was assessed using changes in paw volume and thermal and mechanical pain thresholds of the rats during recovery from anesthesia.

**Results:**

Electroacupuncture at selected auricular acupoints significantly reduced paw edema and mechanical hyperalgesia, with no significant effect on thermal hyperalgesia. The anti-edematous and analgesic effects of AA were abolished by blockade of peripheral cholinergic muscarinic receptors with methyl atropine. Blockade of local muscarinic receptors at the inflamed site with a small dose of atropine also antagonized the anti-edematous effect of AA. By contrast, systemic opioid receptor blockade with naloxone did not antagonize the anti-inflammatory effects of AA.

**Conclusion:**

This study discovers a role of peripheral muscarinic receptors in mediating the anti-inflammatory effects of AA. The cholinergic muscarinic mechanism appears to be more important than the opioid mechanism in the anti-inflammatory action of AA.

## Background

Auricular acupuncture (AA) has been used for a wide varieties of pain conditions, such as cancer pain [1], chronic spinal pain [2,3], phantom limb pain [4], post-operative pain [5] and wound care in patients with burns [6]. Unlike body acupuncture, which has been widely studied for its analgesic mechanisms [7], the mechanism of AA in pain relief remains largely uninvestigated. Since the auricle is innervated by a mix of V, VII, IX and X cranial sensory nerves as well as cervical spinal afferents [8-10] and has central connections distinct from those of body acupoints, the afferent signaling (hence the physiological responses) produced by AA may be substantially different from those produced by body acupuncture.

The anti-inflammatory action of AA is of particular interest as inflammation is a major cause of pain. A cholinergic anti-inflammatory pathway involving activation of vagal efferent nerves was described [11]. Stimulation of the vagus nerve inhibits the development of carrageenan (CA)-induced paw edema and local production of cytokines, and local administration of the vagus nerve neurotransmitter acetylcholine or cholinergic agonists reduces acute inflammation [12]. Acupuncture may activate the cholinergic anti-inflammatory pathway in treatment of inflammatory diseases [11]. Stimulation of auricular afferents excites vagal efferents [13] which may in turn modulate the cholinergic anti-inflammatory pathway. Moreover, opioids were found to be related to the analgesic effects of AA [14].

The present study tests whether auricular acupuncture produces anti-inflammatory effects via opioid and peripheral cholinergic receptors in a rat model.

## Methods

### Rationale

A CA-induced inflammation model responsive to activation of the cholinergic anti-inflammatory pathway [12] was used as previously described [15]. The optimal location for AA, the appropriate intensity of electroacupuncture stimulation and the effects of AA given at different time points were determined with three series of pilot experiments. We then studied the analgesic and anti-edematous effects of AA under the optimal stimulation parameters and examined the antagonistic effects of mascurinic and opioid receptor antagonists.

### Animal treatments

A total of 238 adult male Sprague-Dawley rats weighting 200-260 g were purchased from the Laboratory Animal Services Centre, The Chinese University of Hong Kong and were acclimatized for three days or over with food and water accessible *ad libitum*. The experimental protocols were approved by the Committee on the Use of Human & Animal Subjects in Teaching and Research of the Hong Kong Baptist University. The experiments were carried out in accordance with the Ethical Guidelines for Investigations of Experimental Pain in Conscious Animals published by the International Association for the Study of Pain [16]. Anesthesia was induced with intraperitoneal (i.p.) injection of 400 mg/kg choral hydrate (Fluka, USA), which provided stable anesthesia for 60-80 minutes. In some experiments, animals were kept fully anesthetized with additional anesthetics (20 mg of choral hydrate) when they showed blinking reflex or movements to handling [15]; and in other experiments, animals were allowed to wake up for behavioral testing. The fully anesthetized preparation was used for all the pilot experiments and for Protocol 3 described in the subsection Experimental protocols. Under anesthesia, local inflammation was induced by subcutaneous injection of 50 μl of 2% CA lambda (Sigma-Aldrich, USA) in 0.9% saline at the center of the left paw on the planter surface. At the end of the experiments, animals were sacrificed with overdosed pentobarbital and cervical dislocation.

### Measurements of pain and inflammation

Paw volume was measured at the level of the calcaneus with a plethysmometer (Ugo Basile, Italy) as previously described [15]. Nociception thresholds were evaluated with measurement of thermal and pressure stimuli before anesthesia and at least four hours after the initial anesthetic injection (when the rats had completely recovered from anesthesia and displayed normal walking and exploratory behaviors so that reliable algesic tests could be carried out). Thermal and mechanical hyperalgesia were assessed according to the methods described previously [17,18]. Briefly, for measurement of the latency of paw withdrawal upon thermal stimulation, the animals were placed in a plexiglass enclosure on top of a glass plate and habituated for at least 30 minutes. A thermal stimulator (IITC, USA) was positioned under the glass plate and the focus of the projection bulb was directed to the middle of the plantar surface of the animal with the aid of a mirror attached to the stimulator. The intensity of the light source was adjusted to the level that caused the paw to withdraw at 10-12 seconds in normal rats and a cut-off time of 20 seconds was set so as to prevent tissue damage. The thermal pain threshold was defined as the latency of reflex paw withdrawal. Change in thermal pain threshold was expressed as percentage change in paw withdrawal time from baseline. The paw pressure test was performed with an electronic pressure-meter [19] consisting of a polypropylene pipette tip (diameter: 1 mm) adapted to a hand-held electronic force transducer connected to a data acquisition system (AD Instruments, Australia). Animals were stabilized in a covered acrylic cage with a wire grid floor and the polypropylene tip was applied perpendicularly to each of the five distal footpads with a gradual increase in pressure. The pressure required to induce a flexor response was taken as the pain threshold for each footpad and the mean value of pain thresholds for the five footpads was used as the pressure pain threshold for each animal. Change in pressure pain threshold was expressed as percentage change from the pre-inflammation baseline taken before the induction of anesthesia and CA injection. The assessor who took the above measurements was blinded to the group status of the animal.

### Localization of auricular acupoints

**Figure 1:**
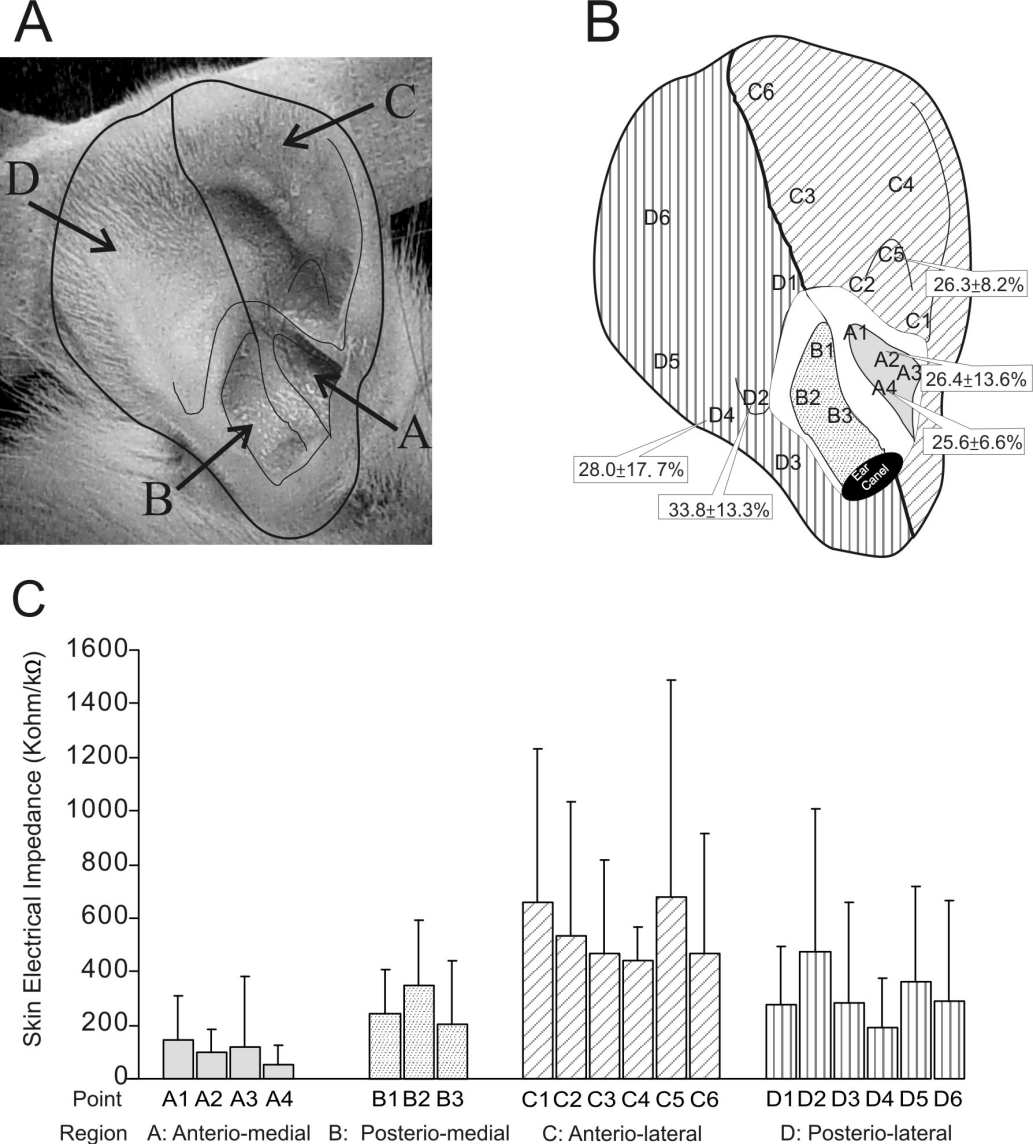
**Illustration of auricular acupoint identification**. A: Photo of the rat right auricle, with lines indicating the anatomical landmarks. B: Drawing of the rat right auricle showing the location of points where electrical impedance was measured. The paw volumes after CA injection and electroacupuncture (0.7-1 mA, 4 Hz) at sites A2, A4, C5, D2 and D5 are shown in the respective boxes, and they are indicative of the anti-inflammatory efficacy of the given site. C: Histograms showing the electrical impedance obtained from the auricle (mean ± SD, *n *= 6).

Auricular acupoints may typically correspond to regions of low electrical impedance [20]. To identify low impedance points, we carried out pilot experiments with six rats according to a method described previously [21]. We divided the rat auricle into four areas based on surface landmarks: anterio-medial (area A), posterio-medial (area B), posterio-lateral (area C) and anterio-lateral (area D) (Figure 1A) corresponding to the cymba conchae, cavita conchae, helix-scapha-antihelix and triangular fossa-crura anthelicis respectively in human. A total of 19 points were identified anatomically according to nearby landmarks and selected for measurements (Figure 1B). A point with impedance lower than 100 kΩ was regarded as a low impedance point [20]. Low impedance points were mainly found in area A (Figure 1C) corresponding to the cymba conchae in human.

To determine the relationship between acupoint location and anti-inflammatory efficacy, we carried out pilot experiments to compare the anti-edematous effects of AA at various auricular points in fully anesthetized animals. AA (0.7-1.0 mA) was applied at one of the five auricular points for 60 minutes after CA injection. The maximal paw volume (mean ± SD) reached at four hours post-CA injection was 26.4 ± 13.6% for A2, 25.6 ± 6.6% for A4, 26.3 ± 8.2% for C5, 28.0 ± 17.7% for D4 and 33.8 ± 13.3% for D2 (*n *= 10 per group; Figure 1B) with the volumes of A2, A4, C5 significantly lower than that of the control (39.8 ± 14.7, *P *< 0.001). Therefore, we selected A4, the point of least edema, for later experiments.

**Figure 2:**
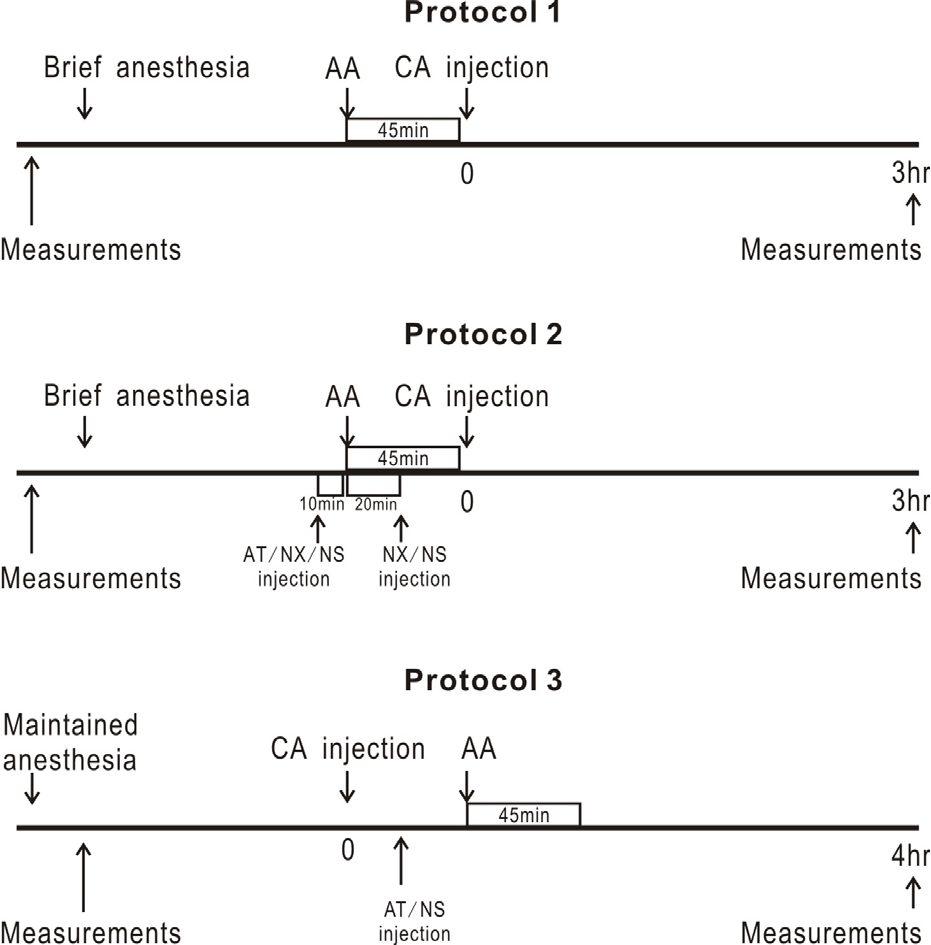
**Schematic drawings showing the time lines for each experimental protocol**. Please note that time indicators are not drawn to scale. In Protocol 2, intraperitoneal injection of receptor antagonists (NX, naloxone; AT, atropine) or the vehicle normal saline (NS) was made 10 minutes before auricular acupuncture (AA) and 20 minutes after AA. In Protocol 3, intraplantar injection of AT/NS was made following CA injection, just before AA.

### AA using electrical stimulation

A pair of stainless steel needles (Carbo, China; 0.2 mm×13 mm) inserted 1 mm deep perpendicularly at A4 bilaterally were connected to an electroacupuncture machine (Cafar Acus II, Sweden) set to deliver 4 Hz rectangular pulses (0.45 ms in duration) in alternating polarity for 45 minutes of stimulation. Pilot experiments were carried out to determine the optimal intensity of stimulation in fully anesthetized animals with paw edema as the outcome measure. Five groups of animals (*n *= 10-12 per group) were given AA at the A4 site at an intensity of either 0 mA (sham electroacupuncture), 0.5 mA, 0.7 mA, 1.0 mA or 1.5 mA one hour after induction of inflammation. The 0.7 mA and 1.0 mA groups had the strongest inhibitory effect on edema. Therefore, for all later experiments, the stimulation intensity was set to 0.7-1.0 mA.

### AA at various time points

A separate series of pilot experiments was carried out to determine if the timing of AA was also important in producing anti-inflammatory effects. Thirty-two (32) rats were randomly divided into four groups (*n *= 8 each) which received either no AA treatment (control) or AA at three time points. AA given either one hour prior to CA injection, immediately after CA injection or one hour after CA injection had similar anti-edematous effects that were significantly different (*P *< 0.05) from that of control. Therefore, in later experiments we applied electroacupuncture either one hour prior to CA injection or immediately after CA injection.

### Receptor blockades

To determine the receptor systems involved in the action of AA, we injected (ip), 10 minutes prior to AA, opioid receptor antagonist naloxone hydrochloride (NX; Sigma, USA; 5 mg/kg in 0.2 ml of saline), the peripheral muscarinic receptor antagonist atropine methyl bromide (AT, Sigma, USA; 2 mg/kg in 0.2 ml of saline) or 0.2 ml of the injection vehicle, namely normal saline (NS). A supplementary dose of NX (2.5 mg/kg in 0.1 ml NS) was given again 30 minutes after the first NX injection [15,22] and the same amount of NS was given to the other groups as control.

In a separate series of experiments, immediately after CA injection, a small dose of AT (12.5 μg in 50 μl of saline) or vehicle was injected intraplantarly at either the left or right paw (Protocol 3 below). All drugs were freshly prepared by a technician unaware of the experimental procedures and the drug identity of the animal group status was revealed to the experimenter only after completion of the testing.

### Experimental protocols

#### Protocol 1

To determine the anti-inflammatory effects of AA, we tested the animals for thermal and pressure pain thresholds and measured paw volumes. They were then anesthetized with choral hydrate and randomly divided into two groups (*n *= 10 per group). One group received AA treatment for 45 minutes while the other received no treatment to serve as control. Then both groups were given CA injection. The animals were fully conscious two hours after anesthesia (at about one hour after CA injection) and thermal and pressure pain thresholds as well as paw volumes were assessed again three hours after CA injection (Figure 2).

#### Protocol 2

To determine the effects of peripheral muscarinic blockade and systemic opioid blockades, we used a 2×3 full factorial design (*n *= 12 per group). On each experimental day six rats were first tested for thermal and pressure pain thresholds and had paw volumes measured. They were then anesthetized and allocated to one of the three pairs that received either injection of naloxone (5 mg/kg ip), methyl-atropine (AT, 2 mg/kg ip) or NS. One rat in each pair was then given AA for 45 minutes 10 minutes after the injection. A second ip injection of NX (2.5 mg/kg) or NS (for the non-NX groups) was given with 30 minutes after the first injection during AA. After AA, all animals were given intraplantar CA injection and thermal and pressure pain thresholds as well as paw volumes were assessed again three hours after CA injection (Figure 2).

#### Protocol 3

To assess the role of local muscarinic receptors, we used paw volumes as the measure of inflammation in fully anesthetized animals. After anesthesia, the paw volumes were measured and CA was injected to induce inflammation. Then animals were divided into three groups (*n *= 8 per group) and received intraplantar injections of (i) AT (12.5 μg in 50 μl of vehicle) at the left (CA-injected) paw with vehicle at the right paw or (ii) AT at the right paw with vehicle at the left paw or (iii) vehicle at both paws. Then AA was given for 45 minutes and the paw volumes were measured at the fourth hour (Figure 2).

### Statistical analysis

Statistical analysis was performed with SPSS (Version 11, SPSS Inc., USA). Paw edema was calculated by the following formula: V = (V_1_/V_0_-1) ×100%, where V is the change in paw volume, V_0 _is the baseline paw volume and V_1 _is the paw volume after induction of inflammation. Changes in thermal and mechanical thresholds were expressed by percentage of the baseline according to this formula: T = T_1_/T_0_, where T is the change in threshold, T_0 _is the baseline threshold reading and T_1 _is the threshold reading after induction of inflammation. Data were expressed as mean ± SD. One-way ANOVA followed by double-sided Dunnett's T *post-hoc *comparison was performed to compare the effects of several experimental groups with the control. Two-way ANOVA followed by Tukey's HSD *post-hoc *comparison was performed to compare the combined effects from AA and receptor antagonists on several indicators of inflammation. Differences were considered statistically significant when *P *< 0.05.

## Results

### Anti-inflammatory effects of AA

**Figure 3:**
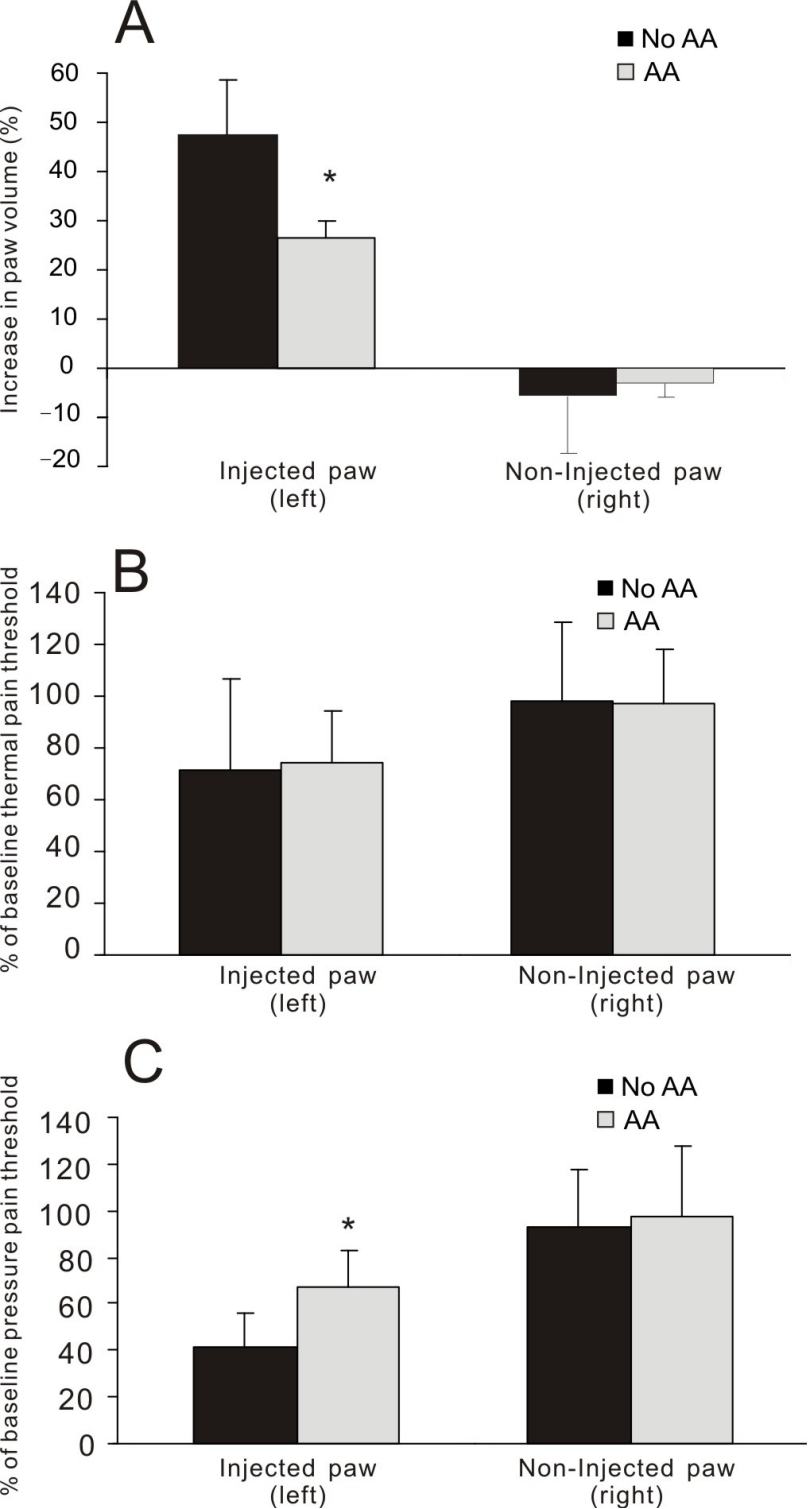
**Histograms showing the effects of auricular acupuncture (AA) on paw volume, thermal pain threshold and pressure pain threshold in two groups of conscious animals (No AA or AA) that had CA-induced inflammation**. Baseline values were obtained before the induction of brief anesthesia and CA injection. Paw volume is expressed as percentage increase from the baseline and thermal or pain threshold is shown as percentage change from the baseline. * *P *< 0.05, comparison between AA and No AA, *n *= 10 per group.

In conscious animals at the third hour post-CA injection (*n *= 10), for the CA-injected paw, the paw volume (mean ± SD) was 47.6 ± 11.1% over the baseline; and the pressure and thermal pain thresholds were significantly reduced to 41.7 ± 14.3% and 71.1 ± 35.5% of the baseline respectively. In the CA-injected paw of animals with AA (*n *= 10), the edema was 26.8 ± 11.0%, significantly less than that of control (*P *< 0.001); and the pressure pain threshold was 67.3 ± 24.3%, significantly higher than that of control (*P *= 0.012). However, there was no significant difference (*P *> 0.05) between control and treatment groups in thermal pain threshold. For the non-injected paw, there was no difference between the baseline and post-CA injection or between the control and treatment groups (Figure 3).

### Effects of peripheral muscarinic blockade or systemic opioid blockade

**Figure 4:**
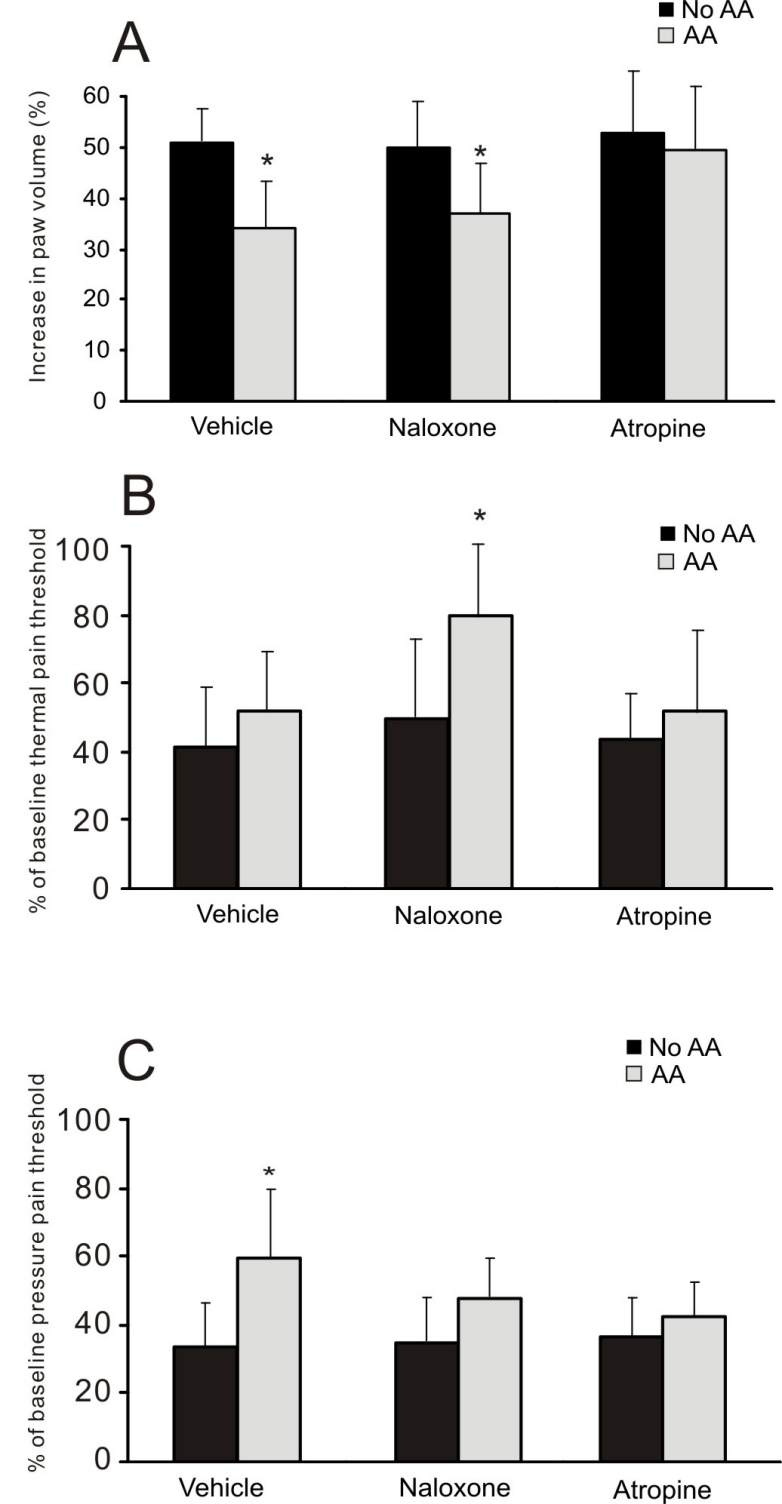
**Histograms showing the effects of methyl atropine (2 mg/kg, ip) and naloxone (5 mg/kg followed by 2.5 mg/kg, ip) on the effects of auricular acupuncture (AA) in conscious animals with CA-induced inflammation**. Baseline values were obtained before the induction of brief anesthesia and CA injection. Paw volume is expressed as percentage increase from the baseline and thermal or pain threshold is shown as percentage change from the baseline. * *P *< 0.05, comparison between AA and No AA with the same ip injection (vehicle, naloxone or atropine), *n *= 12 per group.

A 2×3 full factorial design was used to test whether opioid or peripheral muscarinic receptors were involved in the action of AA (*n *= 12 per group). In the three groups without AA, neither NX nor AT had any effect on edema, thermal or pressure pain threshold in the CA-induced inflammation model (Figure 4). In the AA treatment groups, however, AT antagonized the effects of AA on edema reduction (AA+NS: 33.8 ± 9.12% vs. AA+AT: 49.4 ± 12.2%; *P *= 0.004) and on pressure pain threshold elevation (AA+NS: 50.2 ± 14.9% vs. AA+AT: 35.7 ± 7.8%; *P *= 0.042). By contrast, NX had no effect on the anti-edematous and mechanical hypoalgesic actions of AA (both p > 0.05), but potentiated the analgesic effect of AA on thermal pain threshold (AA+NS: 52.1 ± 18.0% vs. AA+NX: 79.5 ± 24.0%; *P *= 0.018).

### Effect of local muscarinic receptor blockade

**Figure 5:**
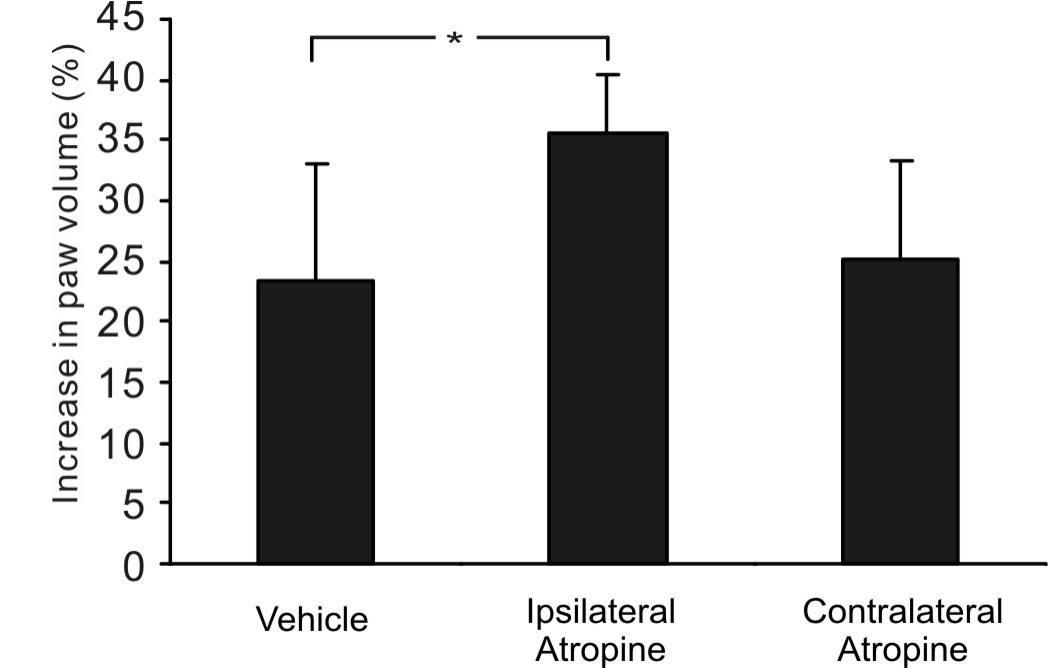
**Histograms showing the effects of local muscarinic receptor blockade on the anti-edematous effect of auricular acupuncture (AA)**. All three groups of animals (*n *= 8 per group) were continuously anesthetized, and received CA-induced inflammation and AA treatment. The first group received intraplantar injections of normal saline (vehicle control) at both paws; the second group was given intraplantar injection of methyl atropine (12.5 μg) ipsilateral to the inflamed side and vehicle injection on the contralateral side; and the third group received the same injection of methyl atropine at the contralateral side, to control for the possible systemic effect of atropine. * *P *< 0.05, *n *= 8 per group.

To further examine the contribution of muscarinic receptors at the site of inflammation, we investigated the effect of local AT administration on the anti-edematous effect of AA in fully anesthetized animals with paw edema as the outcome measure. The group with AT injection at the site of inflammation had significantly higher edema values than the groups with vehicle control injection or contralateral AT injection (Ipsilateral AT: 35.5 ± 4.8% vs. NS-NS: 23.5 ± 9.7% or Contralateral AT: 25.2 ± 8.1%, *n *= 8 per group; *P *= 0.03 between Ipsilateral AT and NS-NS) (Figure 5), indicating that blockade of muscarinic receptors at the site of inflammation reduced the anti-edematous effect of AA.

## Discussion

Using the CA-induced inflammation model, we demonstrate in the present study that peripheral muscarinic receptors, especially those at the site of inflammation, are important in mediating the anti-inflammatory effects of AA. This is in line with the findings that local muscarine application increases mechanical nociceptive thresholds of the skin, which are mediated by M2 receptors [23]. The activation of local M2 receptors leads to nociceptor desensitization by inhibiting the release of calcitonin-gene related peptide (CGRP), a pro-inflammatory neuropeptide in nerve endings [24]. In the formalin model of orofacial nociception in rats, administration of the M2 agonist arecaidine dose-dependently inhibits nocifensive behavior [25]. These findings indicate an important role for muscarinic receptors, especially M2 receptor, in the cholinergic modulation of neurogenic inflammation and in the regulation of nociceptive processing. The present study shows that muscarinic receptors were involved in mediating the anti-edematous and analgesic effects of AA in CA-induced inflammation consisting of both neurogenic and non-neurogenic components [26,27]. It is to be determined, however, which subtypes of muscarinic receptor are involved in mediating effects of AA.

Results of our pilot study (Figure 1) indicated that the anti-inflammatory effect of AA was best evoked from the concha region. This region has preferential innervation from vagal sensory afferents [8,9]. Stimulation of the concha region consistently causes vagal efferent activation, resulting in stomach contraction and bradycardia reversible by atropine [13]. It is assumed that AA also activates vagal efferent nerves in current experiments. However, while evidence suggests that the hindpaw and dorsal root ganglions of the rat has cholinergic receptors [23,28], the signaling pathway between the paw and vagal efferent nerves is to be elucidated.

Consistent with our previous findings [15], the present results clearly shows that neither the anti-edematous effect of AA nor the analgesic effect in mechanical hyperalgesia was antagonized by naloxone. In contrast to muscarinic receptors which play a significant role in mediating both the anti-edematous and analgesic effects of AA, opioid receptors have little role to play in the current anti-inflammatory action of AA.

Our results show that AA attenuated edema and inhibited mechanical hyperalgesia without affecting thermal hyperalgesia. This is consistent with the results of a previous study that electroacupuncture at body acupoints attenuates mechanical hyperalgesia without affecting thermal hyperalgesia in chronic pain due to complete Freund's adjuvant-induced inflammation [29]. The differential effect of AA on mechanical and thermal hyperalgesia may be due to the fact that different receptors and central circuits are responsible for different types of hyperalgesia [30,31].

The present study also found that the thermal pain threshold was significantly increased after AA and administration of naloxone. This was unexpected because neither AA nor naloxone alone altered the thermal pain threshold in the CA-induced inflammation model. While we do not have full explanation for such a phenomenon, we suspect that it may have been caused by a complex interaction between opioids, acetylcholine and other neurochemicals released following AA [32,33]. It is worth noting that naloxone and naltrexone potentiate the analgesic effect of electroacupuncture at body acupoints as measured by tail-flick response to thermal stimulation [34].

When we compared the results from the present study with those from our previous body electroacupuncture study [15], we found that the time course of the anti-edematous effect of electroacupuncture was different. In other words, in the same model of CA-induced inflammation, body electroacupuncture was only effective when given prior to induction of inflammation whereas AA was effective when given either as pre-treatment or post-treatment. CA-induced inflammation starts with a nonphagocytic response occurring in the first 60 minutes after CA injection, followed by a phagocytic response [27]. Our previous study indicates that body electroacupuncture may be effective in inhibiting only the nonphagocytic response, but not the phagocytic response [15]. By contrast, AA may be effective in reducing both nonphagocytic and phagocytic responses, suggesting different mechanisms in body and auricular electroacupuncture.

The stimulation parameters used in the present study provides clues of the fiber classes involved. It was reported that the mean threshold intensities of group II, III and IV fibers in the saphenous nerve were 0.2, 0.5 and 3.0 mA respectively in response to electroacupuncture stimulation of ST36 at 20 Hz, 0.5 ms pulse duration [35,36]. As low frequency (4 Hz) and short pulse duration (0.45 ms) was used in the present study, we expect that few, if any, group IV or C fibers would be excited at 0.7-1.0 mA. In human subjects we found that 0.7-1.0 mA auricular stimulation gave a strong but non-painful stimulation [37]. Taken together, it would be reasonable to assume that the 0.7-1.0 mA stimulation used in our current experiments is a clinically relevant intensity that stimulates mainly mylinated fibers. Further studies are warranted to elucidate the exact neural pathway between auricular afferents and muscarinic receptors at the paw.

Taken together, the present data support the clinical use of auricular acupuncture for pain conditions involving inflammation, and suggest that the therapeutic properties of AA may be different from those of body electroacupuncture.

## Conclusion

This study discovers a role of peripheral muscarinic receptors in mediating the anti-inflammatory effects of AA. The cholinergic muscarinic mechanism appears to be more important than the opioid mechanism in the anti-inflammatory action of AA.

## Abbreviations

AA: Auricular acupuncture; AT: Atropine (Atropine methyl nitrate); CA: Carrageenan (Carrageenan lambda); CGRP: Calcitonin-gene related peptide; ip: intraperitoneal; NS: Normal Saline; NX: Naloxone (Naloxone hydrochloride)

## Competing interests

The authors declare that they have no competing interests.

## Authors' contributions

WYC, HQZ and SPZ conceived and designed the study. SPZ coordinated and WYC carried out the study. WYC and SPZ wrote the manuscript. HQZ provided critical comments on the manuscript. All authors read and approved the final version of the manuscript.
